# Supplementary material for: The interplay between dietary fatty acids and gut microbiota influences host metabolism and hepatic steatosis
Source: Nat Commun. 2023 Sep 1;14:5329. doi: 10.1038/s41467-023-41074-3 (PMC10474162; doi:10.1038/s41467-023-41074-3)
Supplement: Supplementary file 1 — Supplementary Information [file 41467_2023_41074_MOESM1_ESM.pdf]

Supplementary Information

The interplay between dietary fatty acids and gut microbiota influences host metabolism and hepatic steatosis

Schoeler et al.

Supplementary Figure 1	1
Supplementary Figure 2	2
Supplementary Figure 3	3
Supplementary Figure 4	4
Supplementary Figure 5	5
Supplementary Figure 6	6
Supplementary Figure 7	7
Supplementary Figure 8	8
Supplementary Figure 9	9
Supplementary Figure 10	10
Supplementary Table 1	11
Supplementary Table 2	12
Supplementary Table 3	13
Supplementary Table 4	14
Supplementary Table 5	15

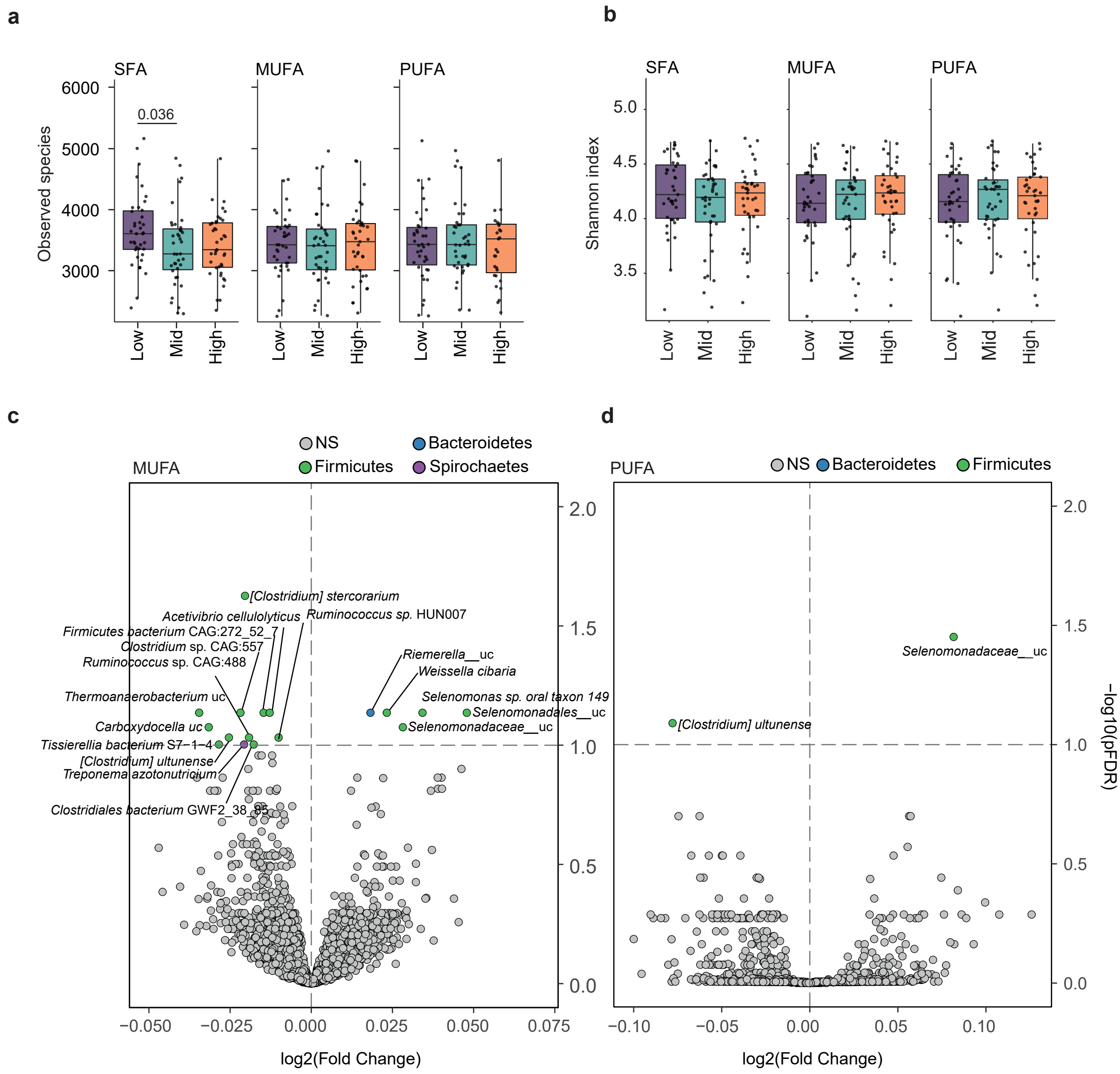

Supplementary Figure 1. The effect of dietary fatty acid saturation level on human fecal microbiota composition. Related to Figure 1. Subjects are from the IRONMET cohort. **a** Number of observed species and the **b** Shannon index in feces of subjects divided into tertiles based on their SFA, MUFA or PUFA consumption. There were no differences in the library size among tertiles. Volcano plots of differential bacterial (pFDR <0.05) associated with the amount of **c** MUFA intake and **d** PUFA intake, after fitting a robust linear regression model to the clr-transformed data controlling for age, sex, BMI and fiber intake. Fold change associated with a unit change in fatty acids and FDR-adjusted values (pFDR) are plotted for each taxon. Significant taxa are colored according to phylum as shown.

n = 117. Significant p-values as determined by two-sided Wilcoxon rank-sum test are displayed in the figure. For box plots in panel a and b: the middle line is the median, the lower and upper hinges are the first and third quartiles, the whiskers extend from the hinge to the largest and smallest value no further than 1.5 × the inter-quartile range (IQR). SFA = saturated fatty acids; MUFA = mono-unsaturated fatty acids; PUFA = poly-unsaturated fatty acids. Source data are provided as a Source Data file.

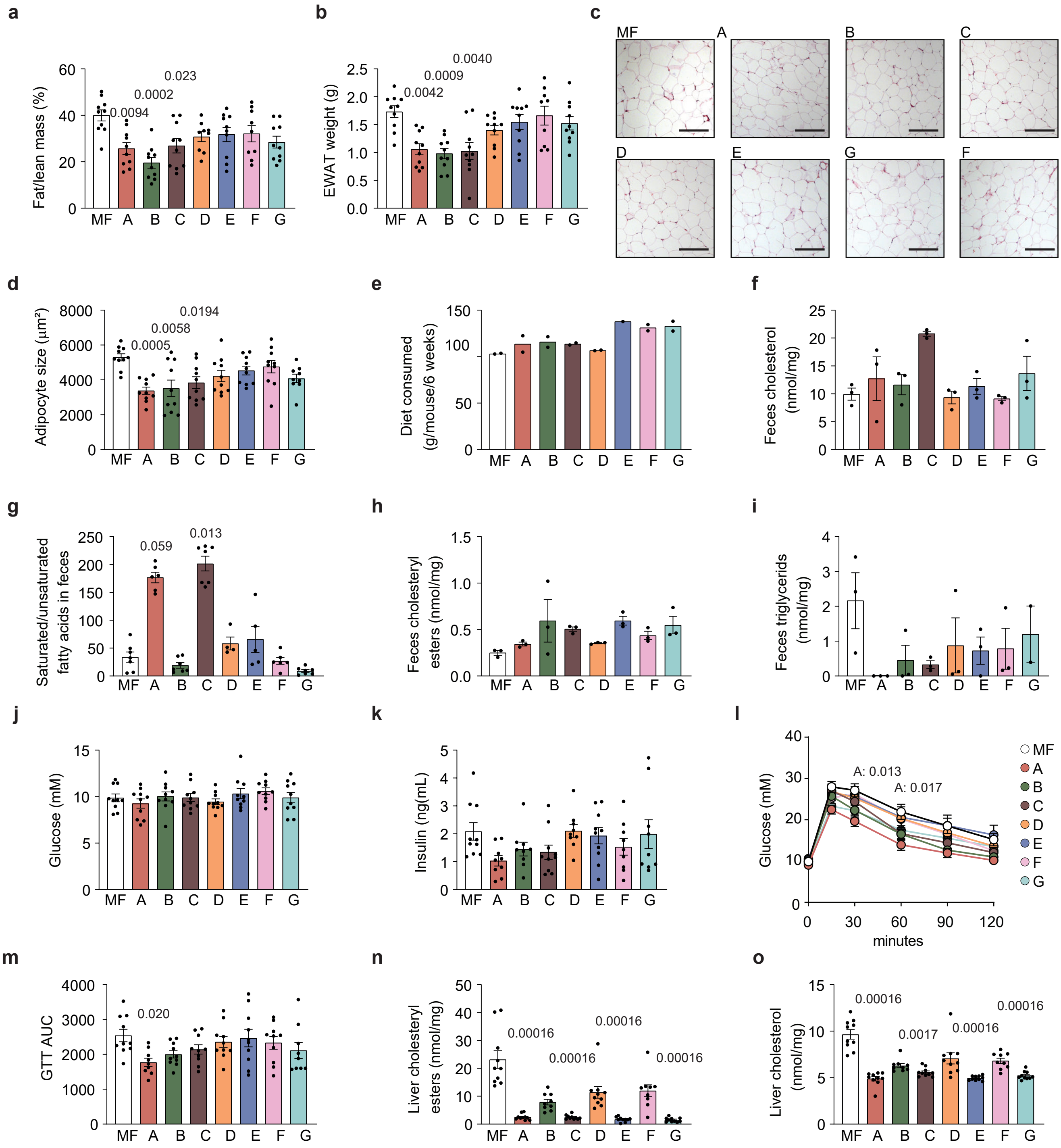

Supplementary Figure 2. Metabolic profile, diet consumption and lipid levels in liver and feces in mice fed high-fat diets. Related to Figure 2. Mice were fed high-fat diets for 9 weeks. **a** Fat/lean mass measured by magnetic resonance imaging during week 9. **b** EWAT weight, **c** hematoxylin and eosin staining of representative WAT tissue (scale bar = 100 µm) and **d** average adipocyte size. **e** Cage-wise measurement of accumulated diet consumption over six weeks (last weeks excluded due to metabolic testing during this period). **f** Fecal cholesterol, **g** ratio between saturated and unsaturated fecal free fatty acids, **h** fecal cholesteryl esters and **i** fecal triglycerides. Fasting **j** glucose and **k** insulin levels. **l-m** Glucose tolerance, **n** liver cholesteryl esters, **o** liver cholesterol.

Panel **a**: *n* = 10 except for D and F where *n* = 9; Panel **b**, **d** and **o**: *n* = 10 except for F where *n* = 9; panel **e**: *n* = 2; panel **f** and **h**: *n* = 3; panel **i**: *n* = 3 except for G where *n* = 2; panel **g**: *n* = 7 (MF, B, C, G), 6 (A, F), 5 (E), 4 (D); panel j: *n* = 10; panel k: *n* = 10 except for A, B , F and G where *n* = 9; panel **l-m**: *n* = 10 except for A and G where *n* = 9. Significant p-values vs MF diet as determined by two-sided Kruskal-Wallis test are displayed in the figure. Data are presented as mean ± SEM. GTT = glucose tolerance test; EWAT = epididymal white adipose tissue. Source data are provided as a Source Data file.

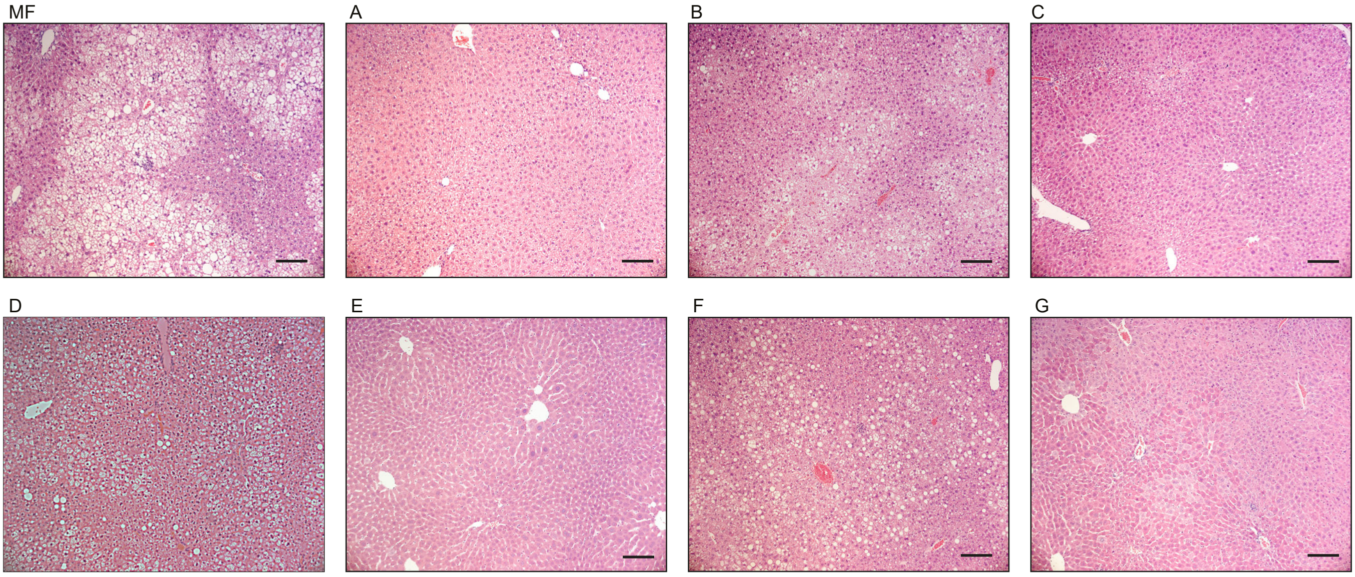

Supplementary Figure 3. Hematoxylin and eosin staining of liver tissue in mice fed high-fat diets. Related to Figure 2. Eight groups of mice were fed high-fat diets with different lipid compositions (MF, A-G) for 9 weeks (scale bar = 100 μm).

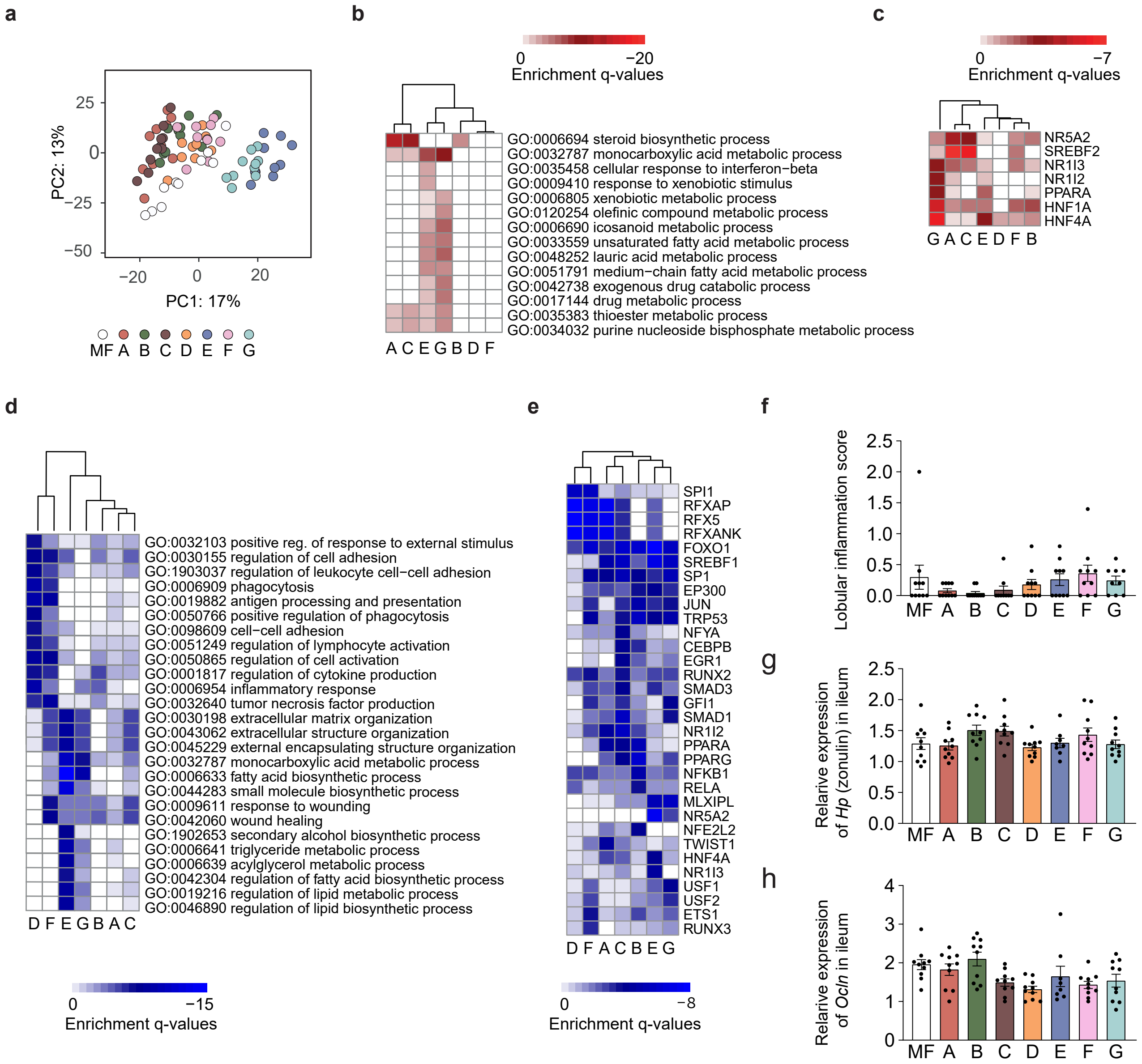

Supplementary Figure 4. Liver phenotype of mice fed high-fat diets. Related to Figure 2. **a** Principal component analysis (PCA) performed on whole hepatic transcriptome. **b** Gene categories enriched in genes upregulated compared to mice fed the MF diet. **c** Transcription factors predicted as up-stream regulators of genes upregulated compared to mice fed MF diet. **d** Gene categories enriched in genes downregulated compared to mice fed MF diet. **e** Transcription factors predicted as up-stream regulators of genes downregulated compared to mice fed the MF diet. **f** Lobular inflammation score. Relative expression of **g** zonulin (*Hp*) and **h** occludin (*Ocln*) in ileum determined by qPCR.

Panel **a**-**e**: n = 10 except for G where n = 9; panel **f**: n = 10 except for G where n = 9; **g**-**h**: n = 10 except for E where n = 8. Data are presented as mean ± SEM.

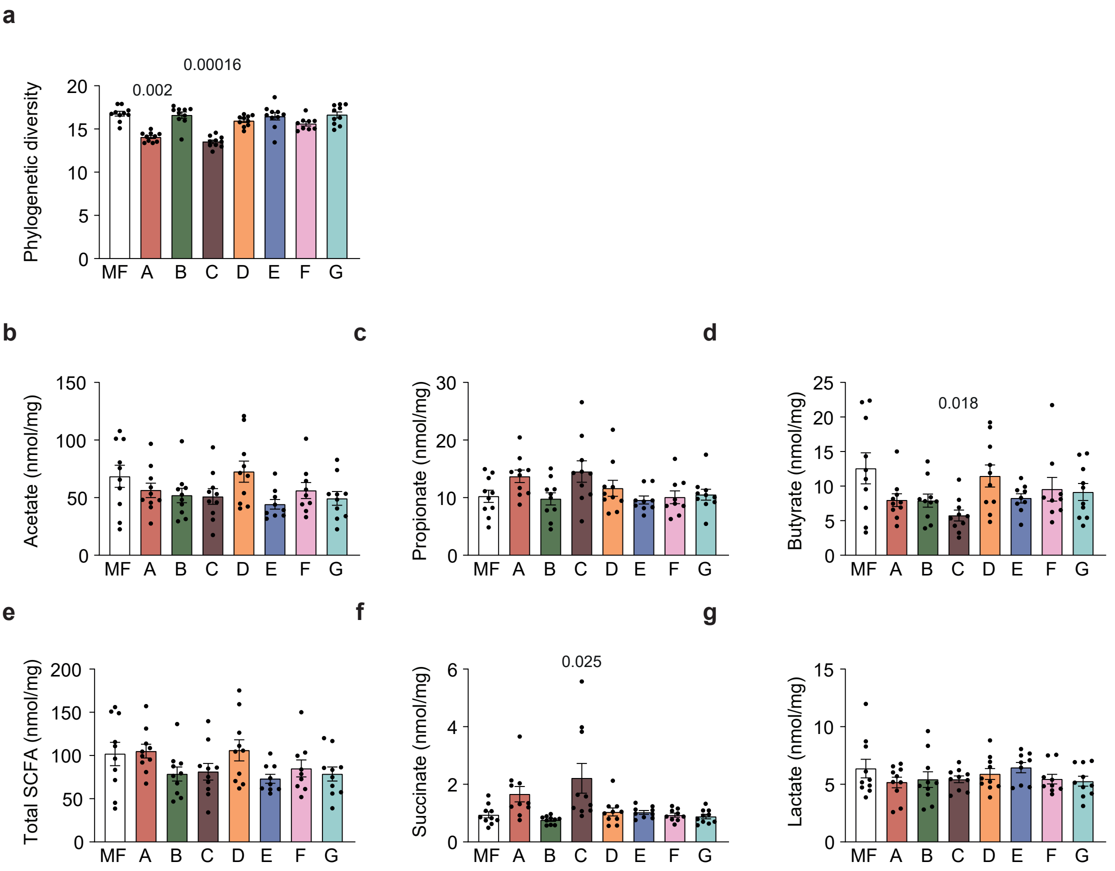

Supplementary Figure 5. Microbiota diversity and cecum levels of SCFA, succinate and lactate in mice fed high-fat diets. Related to Figure 3. **a** Cecum microbiota phylogenetic diversity and concentrations of **b** acetate, **c** propionate, **d** butyrate, **e** total SCFA, **f** succinate and **g** lactate in cecum of mice fed high-fat diets for 9 weeks.

Panel **a**: n = 10 except for F where n = 9; panel **b-g**: n = 10 except for E and F where n = 9. Significant p-values vs MF diet as determined by two-sided Kruskal-Wallis test are displayed in the figure. Data are presented as mean ± SEM. SCFA = short-chain fatty acids. Source data are provided as a Source Data file.

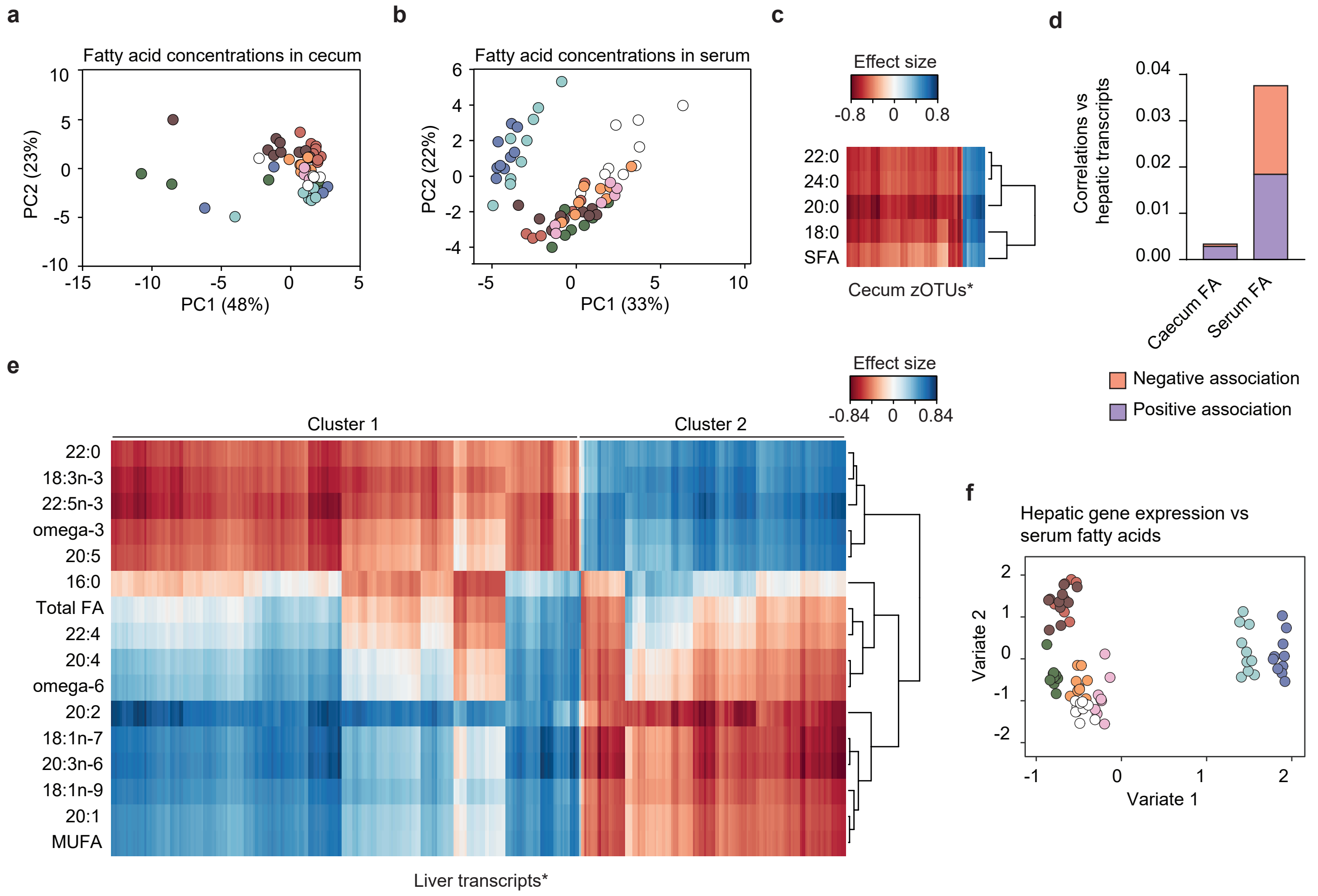

Supplementary Figure 6. Fatty acid composition and correlations between fatty acids, cecum bacteria and hepatic gene expression determined by rCCA analysis. Related to Figure 5. Principal component analysis (PCA) performed on fatty acid concentrations in **a** cecum and **b** serum. **c** Correlations between cecum fatty acids and cecum bacteria (*See bacterial taxa and correlations in Table S4). **d** Density of the correlation network between hepatic gene expression levels, as determined by microarray analysis, and cecal or serum fatty acids determined by regularized canonical correlation analysis (rCCA). **e** Correlations between serum fatty acid levels and liver transcripts (*See Table S6 for GO enrichment of correlated genes). **f** Cluster plot based on correlations between serum fatty acids and hepatic gene expression levels determined by rCCA. Cecum samples: n = 5 (MF), 10 (A), 6 (B), 9 (C), 7 (D), 4 (E), 3 (F), 7 (G); serum samples: n = 9 (MF), 7 (A), 8 (B), 9 (C), 8 (D), 9 (E), 6 (F), 9 (G). Source data are provided as a Source Data file.

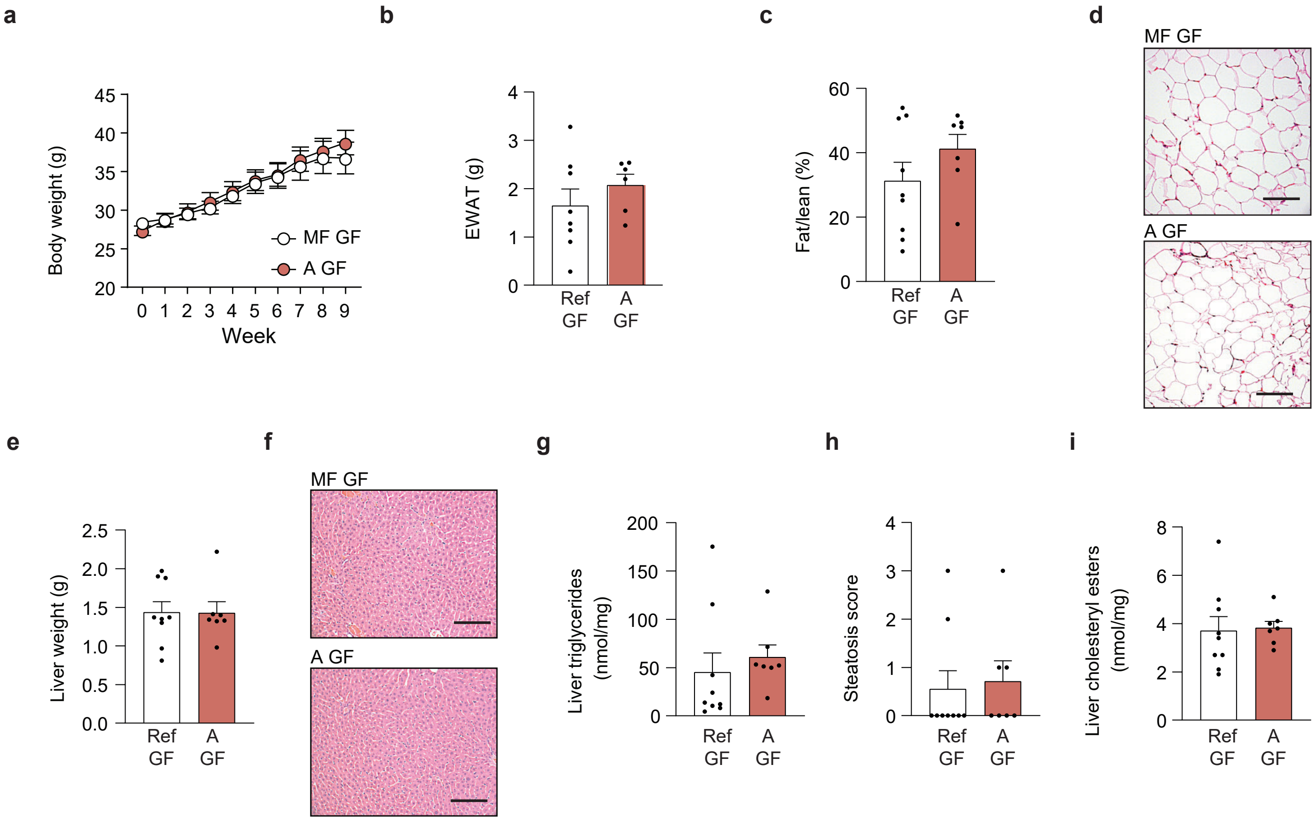

Supplementary Figure 7. Metabolic phenotype of germ-free mice fed MF diet or diet A. Germ-free (GF) mice were fed either MF diet or diet A for 9 weeks. **a** Body weight, **b** EWAT weight, **c** fat/lean mass measured by magnetic resonance imaging at week 9, **d** hematoxylin and eosin staining of representative WAT tissue (scale bar = 100 µm), **e** liver weight, **f** hematoxylin and eosin staining of liver tissue (scale bar = 100 µm), **g** steatosis score, **h** liver triglycerides and **i** liver cholesteryl esters.

n = 9 (MF) and 7 (A). Significance is determined by two-sided Mann–Whitney U test. Data are presented as mean ± SEM. EWAT = epididymal white adipose tissue. Source data are provided as a Source Data file.

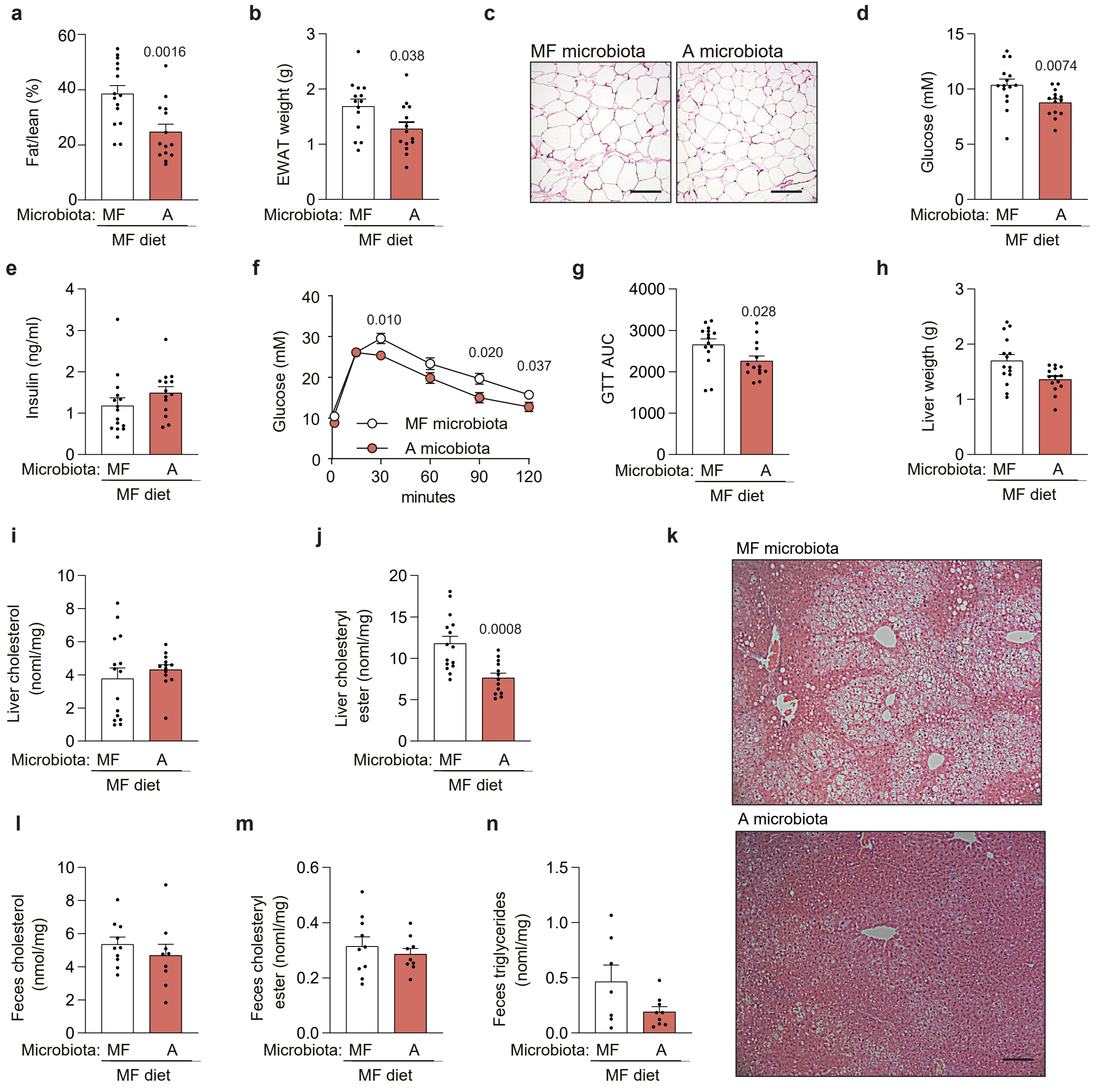

Supplementary Figure 8. Metabolic phenotype, of mice colonized with cecal microbiota from mice fed MF diet or diet A, then subsequently fed MF diet. Related to Figure 6. All mice were fed MF diet for 9 weeks after first microbiota transfer. **a** Fat/lean mass measured by magnetic resonance imaging during week 9 of high-fat diet, **b** EWAT weight, **c** hematoxylin and eosin staining of representative WAT tissue (scale bar = 100 µm), fasting **d** glucose and **e** insulin levels, **f-g** glucose tolerance, **h** liver weight, **i** liver cholesterol, **j** liver cholesteryl esters, **k** hematoxylin and eosin staining of liver tissue (scale bar = 100 µm), **l** fecal cholesteryl esters, **m** fecal triglycerides and **n** fecal free fatty acids.

Panel **a-h**: n = 15 (MF microbiota), 14 (A microbiota) except panel **b** where n = 14 (MF microbiota); panel **i-j**: n = 15 (MF microbiota), 13 (A microbiota); panel l-m: 10 (MF microbiota), 9 (A microbiota); panel **n**: n = 7 (MF microbiota), 9 (A microbiota). Significant p-values determined by two-sided Mann–Whitney U test are displayed in the figure. Data are presented as mean ± SEM. GTT = glucose tolerance test; EWAT = epididymal white adipose tissue. Source data are provided as a Source Data file.

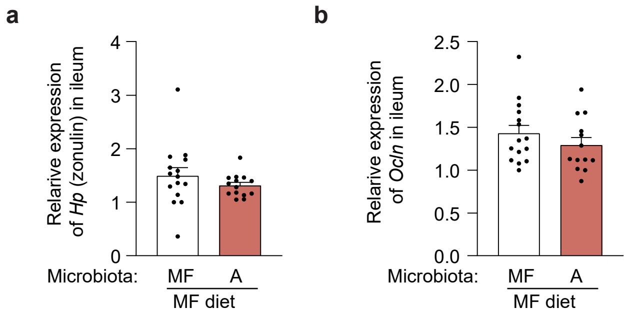

Supplementary Figure 9. Expression of genes encoding tigh junction proteins in mice colonized with cecal microbiota from mice fed either MF diet or diet A, then subsequently fed MF diet. Relative expression of **a** zonulin (*Hp*) (*n* = 15 (MF microbiota) and 13 (A microbiota)) and **b** occludin (*Ocln*) determined by qPCR in ileum.

n = 15 (MF microbiota) and 14 (A microbiota). Data are presented as mean ± SEM. Source data are provided as a Source Data file.

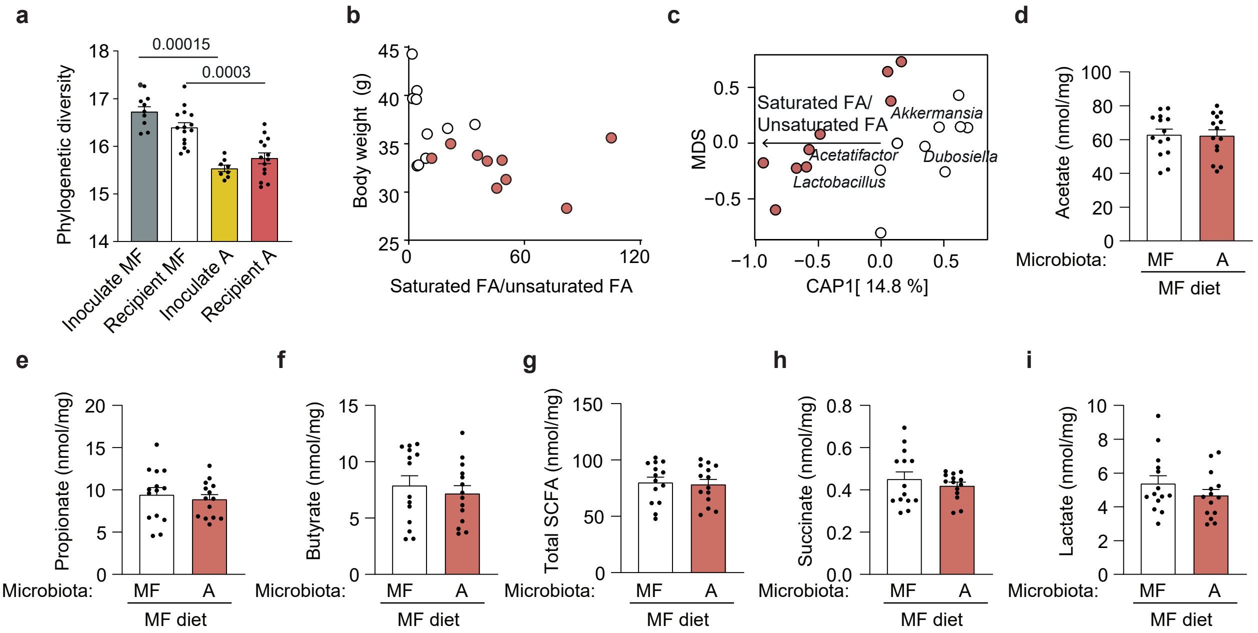

Supplementary Figure 10. Cecum microbiota phylogenetic diversity and metabolites in mice colonized with cecal microbiota from mice fed either MF diet or diet A, then subsequently fed MF diet. Related to Figure 7. **a** Cecum microbiota phylogenetic diversity. **b** Correlation between body weight and ratio between saturated and unsaturated fatty acids. **c** Distance-based redundancy analysis of composition using the ratio of saturated and unsaturated fatty acids in feces. The statistical model constrains 14,8 % of the compositional variation ($p<0.01$). Cecum levels of **d** acetate, **e** propionate, **f** butyrate, **g** total SCFA, **h** succinate and **i** lactate.

Panel **a**: n = 9 (MF microbiota inoculate), 15 (MF microbiota recipients), 8 (A microbiota inoculate), 14 (A microbiota recipients); panel **b**: n = 10 (MF microbiota) and 9 (A microbiota); panel **d-h**: n = 14. Significant p-values determined by two-sided Mann–Whitney U test are displayed in the figure. Data are presented as mean ± SEM. SCFA = short-chain fatty acids. Source data are provided as a Source Data file.

Supplementary Table 1. GO enrichment analysis of genes contributing to Variate 1 and 2 in sPLS plot. Related to Figure 2j.

Pathway	GO	FDR	Fold Enrichment
**sPLS Vatiate 1**			
Fatty acid biosynthetic process	0006633	5.18E-05	24.65
Monocarboxylic acid metabolic process	0032787	9.93E-05	8.75
Triglyceride metabolic process	0006641	0.000142	27.63
Anion transport	0006820	0.000834	7.45
Small molecule biosynthetic process	0044283	0.000834	7.54
Positive regulation of fatty acid biosynthetic process	0045723	0.000834	72.95
**sPLS Variate 2**			
Small molecule metabolic process	0044281	2.16E-05	7.13
Sterol biosynthetic process	0016126	3.39E-05	85.3
Organic hydroxyl-compound metabolic process	1901615	0.000338	13.1
Secondary alcohol metabolic process	1902652	0.000338	33.1
Steroid biosynthetic process	0006694	0.000442	28.7
Small molecule biosynthetic process	0044283	0.000522	10.7

Supplementary Table 2. Cytokines in serum from *vena cava.* Eight groups of mice (MF, A-G) were fed high fat diets with different lipid compositions for 9 weeks. n = 10 except for F where n is 9. *p = 0.01 vs MF diet as determined by Kruskal-Wallis test. Data are presented as mean ± SEM. Source data are provided as a Source Data file.

	MF	A	B	C	D	E	F	G
**IFN-γ**	0,37 ± 0,087	0,28 ± 0,063	0,39 ± 0,046	0,44 ± 0,11	0,33 ± 0,067	0,77 ± 0,18	0,52 ± 0,12	0,87 ± 0,14*
**Il10**	9,8 ± 1,2	8,7 ± 0,83	7,5 ± 0,88	11 ± 2	6,2 ± 0,71	9,9 ± 1,1	9,1 ± 1	8,9 ± 1,2
**Il12p70**	17 ± 10	7,8 ± 2,8	5 ± 1,7	13 ± 8,7	10 ± 4,2	4 ± 2	52 ± 34	10 ± 2,9
**Il13**	0.15 ± 0.15	0 ± 0	0 ± 0	1.2± 0.77	0.22 ± 0.20	0.35 ± 0.29	1.2 ± 1.0	0.0 ± 0.0
**IL-1β**	1,4 ± 0,27	0,23 ± 0,1	2,3 ± 1,4	0,53 ± 0,34	0,45 ± 0,17	4,6 ± 3,2	0,43 ± 0,21	0,36 ± 0,13
**Il5**	4 ± 1,2	2 ± 0,3	2,8 ± 0,47	1,8 ± 0,21	2 ± 0,23	2,6 ± 0,19	2,7 ± 0,31	3,4 ± 0,8
**KC**	85 ± 10	88 ± 19	78 ± 7,4	73 ± 9	88 ± 13	94 ± 9,1	96 ± 10	79 ± 3,8
**TNFα**	9,8 ± 0,66	7,5 ± 0,49	9 ± 0,49	8,2 ± 0,68	8,1 ± 1	10 ± 0,73	9,9 ± 0,51	9,8 ± 0,88
**Il2**	0,73 ± 0,14	0,89 ± 0,16	0,55 ± 0,11	0,87 ± 0,12	0,81 ± 0,1	0,79 ± 0,21	1,1 ± 0,35	0,77 ± 0,2
**Il4**	0,37 ± 0,24	0,089 ± 0,039	0,05 ± 0,021	0,1 ± 0,068*	0,076 ± 0,029	0,024 ± 0,022	0,29 ± 0,22	0,096 ± 0,041

Supplementary Table 3. Pairwise analysis of dietary groups (anosim, 9999 permutations) based on Bray-Curtis dissimilarity or UniFrac distance. Related to Figure 3a,b.

	**Bray Curtis**		**Unweighted UniFrac**	
**Test variables**	**R value**	**P value**	**R value**	**P value**
**MF vs A**	0.73	0.0001	0.99	0.0001
**MF vs B**	0.38	0.0016	0.36	0.0002
**MF vs C**	0.87	0.0001	0.97	0.0001
**MF vs D**	0.06	0.1151	0.24	0.0005
**MF vs E**	0.17	0.0084	0.32	0.0001
**MF vs F**	0.14	0.0269	0.59	0.0001
**MF vs G**	0.26	0.0073	0.26	0.0010
**A vs B**	0.82	0.0001	0.96	0.0001
**A vs C**	0.24	0.0012	0.25	0.0003
**A vs D**	0.69	0.0001	0.89	0.0001
**A vs E**	0.55	0.0001	0.98	0.0001
**A vs F**	0.74	0.0001	0.99	0.0001
**A vs G**	0.55	0.0001	0.99	0.0001
**B vs C**	0.85	0.0001	0.97	0.0001
**B vs D**	0.50	0.0002	0.55	0.0001
**B vs E**	0.44	0.0004	0.22	0.0001
**B vs F**	0.28	0.0061	0.62	0.0001
**B vs G**	0.62	0.0001	0.38	0.0001
**C vs D**	0.83	0.0001	0.87	0.0001
**C vs E**	0.63	0.0001	0.99	0.0001
**C vs F**	0.81	0.0001	0.99	0.0001
**C vs G**	0.66	0.0001	0.99	0.0001
**D vs E**	0.30	0.0001	0.44	0.0001
**D vs F**	0.12	0.0547	0.35	0.0001
**D vs G**	0.40	0.0004	0.38	0.0001
**E vs F**	0.24	0.0024	0.58	0.0001
**E vs G**	0.04	0.1911	0.25	0.0009
**F vs G**	0.41	0.0008	0.41	0.0002

Supplementary Table 4. GO enrichment analysis of hepatic genes correlating with serum fatty acid levels in Cluster 1 and Cluster 2. Related to Supplementary Figure 6e.

Pathway	GO	FDR	Fold Enrichment
**Cluster 1**			
Lipid metabolic process	6629	3.55E-31	5.97
Small molecule metabolic process	44281	2.67E-27	4.82
Steroid metabolic process	8202	1.15E-24	12.95
Steroid biosynthetic process	6694	1.63E-22	18.82
Organic hydroxy compound biosynthetic process	1901617	1.63E-22	14.93
Organic hydroxy compound metabolic process	1901615	2.97E-20	8.12
Monocarboxylic acid metabolic process	32787	2.95E-17	6.82
Regulation of lipid metabolic process	19216	3.60E-12	7.75
Neutral lipid metabolic process	6638	1.26E-10	12.48
Unsaturated fatty acid metabolic process	33559	6.73E-10	12.52
Thioester metabolic process	35383	3.09E-08	13.92
Regulation of small molecule metabolic process	62012	5.76E-08	6.21
Ribose phosphate metabolic process	19693	3.07E-07	5.56
Sulfur compound metabolic process	6790	7.31E-07	6.00
Organic hydroxy compound transport	15850	7.82E-07	6.46
Regulation of cellular ketone metabolic process	10565	8.46E-07	9.99
Lipid transport	6869	1.58E-06	5.25
Nucleobase-containing small molecule metabolic process	55086	1.58E-06	4.43
Regulation of plasma lipoprotein particle levels	97006	2.78E-06	15.10
Purine-containing compound metabolic process	72521	3.17E-06	4.98
Regulation of transport	51049	8.34E-06	2.46
Cellular ketone metabolic process	42180	1.06E-05	6.80
Homeostatic process	42592	1.13E-05	2.46
Carbohydrate derivative metabolic process	1901135	2.56E-05	3.06
Response to nitrogen compound	1901698	2.96E-05	2.80
Cellular lipid catabolic process	44242	7.02E-05	6.11
Regulation of cholesterol esterification	10872	7.13E-05	39.65
Response to nutrient levels	31667	7.13E-05	3.86
**Cluster 2**			
Long-chain fatty acid metabolic process	1676	2.02E-16	29.00
Lipid metabolic process	6629	2.17E-16	5.78
Monocarboxylic acid metabolic process	32787	3.53E-15	8.88
Small molecule metabolic process	44281	1.31E-12	4.41
Olefinic compound metabolic process	120254	5.84E-07	13.11
Icosanoid metabolic process	6690	1.57E-06	14.00
Thioester metabolic process	35383	1.57E-06	17.74
Xenobiotic metabolic process	6805	2.83E-06	16.35
Response to xenobiotic stimulus	9410	9.14E-06	13.90

Supplementary Table 5. Cytokines in serum from *vena cava* mice transplanted with caecum microbiota from mice fed MF or A diet and subsequently fed MF diet for 9 weeks. n = 15 (MF microbiota), 14 = (A microbiota). Data are presented as mean ± S.E.M. Source data are provided as a Source Data file.

	MF microbiota	A microbiota
**IFN-γ**	0,49 ± 0,19	0,46 ± 0,23
**IL-10**	8,0 ± 3,3	4,5 ± 0,49
**IL-12p70**	5,9 ± 2,9	5,3 ± 2,4
**IL-13**	0,49 ± 0,38	0,078 ± 0,075
**IL-1β**	ND	ND
**IL-2**	0,32 ± 0,068	0,59 ± 0,081
**IL-4**	0,084 ± 0,061	0,062 ± 0,036
**IL-5**	1,9 ± 0,26	1,8 ± 0,23
**KC/GRO**	73 ± 7,9	65 ± 9,1
**TNF-α**	7,0 ± 0,67	8,2 ± 1,7
