# Supplementary material for: The interplay between dietary fatty acids and gut microbiota influences host metabolism and hepatic steatosis
Source: Nat Commun. 2023 Sep 1;14:5329. doi: 10.1038/s41467-023-41074-3 (PMC10474162; doi:10.1038/s41467-023-41074-3)
Supplement: Supplementary file 3 — Description of supplementary files [file 41467_2023_41074_MOESM3_ESM.pdf]

Supplementary Data Legends

File name: Supplementary Data 1
Description: Correlation coefficients between cecal fatty acids and bacterial OTUs derived from rCCA analysis. Significant correlations are highlighted in grey ($R>0.265$, $p<0.05$ for $n=56$). Related to Figure 5a,b and Extended Data Figure 6a.

File name: Supplementary Data 2.
Description: Positive and negative significant interactions in network analysis between cecum fatty acids, caeum bacteria and host metabolic features. Related to Figure 5c.
