# Supplementary material for: The interplay between dietary fatty acids and gut microbiota influences host metabolism and hepatic steatosis
Source: Nat Commun. 2023 Sep 1;14:5329. doi: 10.1038/s41467-023-41074-3 (PMC10474162; doi:10.1038/s41467-023-41074-3)
Supplement: Supplementary file 6 — Reporting Summary [file 41467_2023_41074_MOESM6_ESM.pdf]

nature portfolio

Corresponding author(s): Robert Caesar

Last updated by author(s): Aug 8, 2023

Reporting Summary

Nature Portfolio wishes to improve the reproducibility of the work that we publish. This form provides structure for consistency and transparency in reporting. For further information on Nature Portfolio policies, see our Editorial Policies and the Editorial Policy Checklist.

Statistics

For all statistical analyses, confirm that the following items are present in the figure legend, table legend, main text, or Methods section.

n/a	Confirmed	
☐	☒	The exact sample size (*n*) for each experimental group/condition, given as a discrete number and unit of measurement
☐	☒	A statement on whether measurements were taken from distinct samples or whether the same sample was measured repeatedly
☐	☒	The statistical test(s) used AND whether they are one- or two-sided<br>*Only common tests should be described solely by name; describe more complex techniques in the Methods section.*
☐	☒	A description of all covariates tested
☐	☒	A description of any assumptions or corrections, such as tests of normality and adjustment for multiple comparisons
☐	☒	A full description of the statistical parameters including central tendency (e.g. means) or other basic estimates (e.g. regression coefficient) AND variation (e.g. standard deviation) or associated estimates of uncertainty (e.g. confidence intervals)
☐	☒	For null hypothesis testing, the test statistic (e.g. *F*, *t*, *r*) with confidence intervals, effect sizes, degrees of freedom and *P* value noted<br>*Give P values as exact values whenever suitable.*
☒	☐	For Bayesian analysis, information on the choice of priors and Markov chain Monte Carlo settings
☒	☐	For hierarchical and complex designs, identification of the appropriate level for tests and full reporting of outcomes
☐	☒	Estimates of effect sizes (e.g. Cohen's *d*, Pearson's *r*), indicating how they were calculated

Our web collection on statistics for biologists contains articles on many of the points above.

Software and code

Policy information about availability of computer code

Data collection	No softwares were used.
Data analysis	Prism (version 9.1.2) Graphpad https://www.graphpad.com/<br>Biopix iQ Software (v. 2.1.3) Biopix<br>R (v.3.1.0) software (R Core Team, 2018)<br>https://www.r-project.org/<br>prinseq-lite-0.20.4 (Schmieder and Edwards, 2011)<br>https://github.com/uwb-linux/prinseq<br>bowtie 2-2.3.4.315 (Langmead and Salzberg, 2012)<br>http://bowtie-bio.sourceforge.net/bowtie2/index.shtml<br>Kaiju v1.6.220 (Menzel et al., 2016)<br>https://github.com/bioinformatics-centre/kaiju<br>zCompositions v.14.0-1 (Palarea-Albaladejo and Martín-Fernández, 2015)<br>https://github.com/Japal/zCompositions<br>“compositions” (van den Boogaart and Tolosana-Delgado, 2008)<br>http://www.stat.boogaart.de/compositions/<br>Limma (v.3.30.13) (Smyth, 2004)<br>http://bioinf.wehi.edu.au/limma<br>SGoF (v2.3.2) (Carvajal-Rodríguez et al., 2009)

https://github.com/cran/sgof
phyloseq v.1.28 (McMurdie and Holmes, 2013)
https://github.com/joey711/phyloseq
metadeconfoundR (v. 0.2.6)
https://github.com/TillBirkner/metadeconfoundR
ggplot2 (v.3.3.3) (Wickham, 2016)
https://ggplot2.tidyverse.org/
Picante (v1.8.2-package) (Kembel et al., 2010)
https://github.com/skembel/picante
vegan (v.2.5-6) R package (Oksanen et al., 2013)
https://github.com/vegandevs/vegan
rtk (v. 0.2.6.1) (Saary et al., 2017)
https://github.com/SimonRit/RTK
MAFFT (v.7.407) (Katoh and Standley, 2013)
https://mafft.cbrc.jp/alignment/software/
FastTree (v.2.1.10) (Price et al., 2009)
http://www.microbesonline.org/fasttree/
Usearch (v.11) (Edgar, 2010)
https://www.drive5.com/usearch/
Bioconductor packages v3.0 (Gentleman et al., 2004)
http://www. bioconductor.org
Revigo (v.1.8.1) (Supek et al., 2011)
http://revigo.irb.hr/
Enrichr (v.2.1) (Kuleshov et al., 2016)
https://maayanlab.cloud/Enrichr/
mixOmics (v6.10.9) (Rohart et al., 2017)
http://mixomics.org/

For manuscripts utilizing custom algorithms or software that are central to the research but not yet described in published literature, software must be made available to editors and reviewers. We strongly encourage code deposition in a community repository (e.g. GitHub). See the Nature Portfolio guidelines for submitting code & software for further information.

Data

Policy information about availability of data

All manuscripts must include a data availability statement. This statement should provide the following information, where applicable:

- Accession codes, unique identifiers, or web links for publicly available datasets
- A description of any restrictions on data availability
- For clinical datasets or third party data, please ensure that the statement adheres to our policy

The raw metagenomic sequence data of the 114 human subjects from the Ironmet cohort have been deposited in the European Nucleotide Archive (ENA) under the project number PRJEB39631 with the accession numbers ERS4859818-ERS4859933 (https://www.ebi.ac.uk/ena/browser/view/PRJEB39631).
The microarray data generated in this study have been deposited in NCBI GEO under accession code GSE222060 (https://www.ncbi.nlm.nih.gov/geo/query/acc.cgi?acc=GSE222060).
The mouse cecum 16S rRNA data generated in this study have been deposited in the European Nucleotide Archive (ENA) under accession code PRJEB58626 (https://www.ebi.ac.uk/ena/browser/view/PRJEB58626).
Data generated in this study are provided in the Source Data file.

Research involving human participants, their data, or biological material

Policy information about studies with human participants or human data. See also policy information about sex, gender (identity/presentation), and sexual orientation and race, ethnicity and racism.

Reporting on sex and gender	Subjects from the human cohort (n = 117; 79 women, 35 men).<br>Sex and gender were considered in the study design, all subjects were analyzed together.<br>Sex was determined based on self-report.<br>No sex- and gender-based analyses have been performed a priori and we have not performed adhoc analysis due to low number of replicates.
Reporting on race, ethnicity, or other socially relevant groupings	No data on race, ethnicity, or other socially relevant groupings was collected.
Population characteristics	Population characteristics has been previously described in Arnoriaga-Rodriguez et al. (https://gut.bmj.com/content/70/12/2283#T1). Consecutive middle-aged subjects, 27.2–66.6 years, were included. Patients with obesity (body mass index (BMI) ≥30 kg/m2) and age-matched and sex-matched subjects without obesity (BMI 18.5–<30 kg/m2), were eligible. Exclusion criteria were type 2 diabetes mellitus, chronic inflammatory systemic diseases, acute or chronic infections in the previous month; use of antibiotic, antifungal, antiviral or treatment with proton pump inhibitors; severe disorders of eating behaviour or major psychiatric antecedents; neurological diseases, history of trauma or injured brain, language

	disorders and excessive alcohol intake (≥40 g OH/day in women or 80 g OH/day in men).
Recruitment	Subjects from the human cohort (n = 117; 79 women, 35 men) were recruited at the Endocrinology Department of Dr. Josep Trueta University Hospital, Girona, Spain. The recruitment of subjects started in January 2016 and finished in October 2017. Consecutive middle-aged subjects, 27.2–66.6 years, were included. Subjects with obesity (body mass index (BMI) ≥ 30 kg/m2, n=65) and age-matched and sex-matched subjects without obesity (BMI 18.5–30 kg/m2, n=52) were eligible.
Ethics oversight	The Institutional Review Board - Ethics Committee and the Committee for Clinical Research of Dr. Josep Trueta University Hospital (Girona, Spain) approved the study protocol and informed written consent was obtained from all participants.

Note that full information on the approval of the study protocol must also be provided in the manuscript.

Field-specific reporting

Please select the one below that is the best fit for your research. If you are not sure, read the appropriate sections before making your selection.

☒ Life sciences ☐ Behavioural & social sciences ☐ Ecological, evolutionary & environmental sciences

For a reference copy of the document with all sections, see nature.com/documents/nr-reporting-summary-flat.pdf

Life sciences study design

All studies must disclose on these points even when the disclosure is negative.

Sample size	The human study was performed on a cohort that was not a priori designed for the current study but where metagenome data, diet consumption and liver status were measured. This made it useful for the purpose of the study but did not allow selection of a sample size. Moreover, the primary outcome in the human study is microbiome composition which is composed of complex data which do not allow sample size calculation. Hence, no power calculation was performed.<br>No power calculations were performed for the mouse studies. The number of replicates was based on experience from previous studies with similar design performed in our lab (https://www.sciencedirect.com/science/article/pii/S1550413115003897?via%3Dihub). The micobiota transfer experiment had 15 instead of 10 replicates since the experimental design may cause loss of mice and/or increased variance in outcome data.
Data exclusions	No data were excluded from the human study.<br>In mouse experiments data points are missing due to loss samples, technical issues or other reasons. The reasons are stated along with the data in the Source data file.
Replication	The metabolic profile of mice fed MF and A were confirmed in a follow-up independent experiment (data not included in ms). All data obtained from this experiment replicated the results from the initial experiment.<br>Since the human study contained unique material and the mouse studies were very large no other experiments were replicated.
Randomization	The design of the human study was cross-sectional and no prior division into groups was performed.<br>Mice were divided into groups based on starting weight at the beginning of the experiment.
Blinding	The investigators performing data collection in the human study did not have knowledge of the research question of the present study. For practial reasons analysis of data from the human study was not perform blinded.<br>For practical reasons, it was impossible to perform mouse experiments and most downstream analyses blinded.<br>The exceptions from this were lipid analysis and evaluation of histology that were performed blinded.

Reporting for specific materials, systems and methods

We require information from authors about some types of materials, experimental systems and methods used in many studies. Here, indicate whether each material, system or method listed is relevant to your study. If you are not sure if a list item applies to your research, read the appropriate section before selecting a response.

Materials & experimental systems

n/a	Involved in the study	
☒	☐	Antibodies
☒	☐	Eukaryotic cell lines
☒	☐	Palaeontology and archaeology
☐	☒	Animals and other organisms
☒	☐	Clinical data
☒	☐	Dual use research of concern
☒	☐	Plants

Methods

n/a	Involved in the study	
☒	☐	ChIP-seq
☒	☐	Flow cytometry
☒	☐	MRI-based neuroimaging

Animals and other research organisms

Policy information about studies involving animals; ARRIVE guidelines recommended for reporting animal research, and Sex and Gender in Research

Laboratory animals	C57Bl/6 mice were maintained at a temperature of 20 ± 1°C, an air humidity of 45-70% at a 12-h light/dark cycle (light from 7 am to 7 pm) under standard specific-pathogen-free (SPF) or GF conditions. All mice were males, 8 weeks old and weight matched at the start of the experiments. Mice were fed irradiated isocaloric diets differing only in their composition of fat (SPF mice: 10 mice per group, 5 mice per cage, 2 cages per group, in total 80 mice (1 mouse died); GF mice: 9 or 7 mice per group, 2-5 mice per cage, 2 cages per group, in total 16 mice; Envigo TD.180342, TD.180343, TD.180344, TD.180345, TD.180346, TD.180347, TD.180348, TD.180349; 42% kcal fat, 0.2% weight total cholesterol; Figure 2A-B), for 9 weeks.
Wild animals	No wild animals were used
Reporting on sex	Only male mice were used
Field-collected samples	No field collected samples were used in the study.
Ethics oversight	All experiments were performed with protocols approved by the University of Gothenburg Animal Studies Committee.

Note that full information on the approval of the study protocol must also be provided in the manuscript.
